# Assessment and comparison of thermochemical pathways for the rice residues valorization: pyrolysis and gasification

**DOI:** 10.1007/s11356-024-32241-0

**Published:** 2024-02-06

**Authors:** Myriam Quintero-Naucil, Jairo Salcedo-Mendoza, Juan Camilo Solarte-Toro, Valentina Aristizábal-Marulanda

**Affiliations:** 1 Facultad de Ingeniería, Grupo Procesos Agroindustriales y Desarrollo Sostenible (PADES), Universidad de Sucre, Sincelejo, Colombia; 2 Grupo de Investigación en Procesos Químicos, Catalíticos y Biotecnológicos, Instituto de Biotecnología y Agroindustria, Universidad Nacional de Colombia – Sede Manizales, Manizales, Colombia; 3 Facultad de Tecnologías, Escuela de Tecnología Química, Grupo de Investigación en Desarrollo de Procesos Químicos, Universidad Tecnológica de Pereira, Carrera 27 #10-02 Álamos, Block 6, 660003 Pereira, Colombia

**Keywords:** Rice husk, Rice straw, Fast pyrolysis, Gasification, Biorefinery scenarios, Techno-economic, Energy and environmental assessment

## Abstract

Lignocellulosic biomass conversion applying thermochemical routes has been postulated as an alternative for generating renewable energy. This research compares energy-driven biorefineries based on two thermochemical routes addressed to upgrade rice husk and rice straw produced in the Department of Sucre-Colombia. Initially, this research analyzes the physico-chemical and structural characterization of the rice residues. Four different scenarios were proposed to compare the energy-driven biorefineries based on fast pyrolysis and gasification considering technical, economic, and environmental metrics. These biorefineries were simulated using the Aspen Plus V.14.0 software. The novelty of this research is focused on the identification of the biorefinery with the best techno-economic, energetic, and environmental performance in the Colombian context. Economic and environmental analyses were done by using economic metrics and emissions. From an economic perspective, the stand-alone gasification process did not have a positive economic margin. In contrast, the fast pyrolysis process has the best economic performance since this process has a positive profit margin. Indeed, scenario 1 (fast pyrolysis of both rice residues) presented an economic margin of 13.75% and emissions of 2170.92 kgCO_2_eq/kg for 10 years. However, this scenario was not energetically the best, holding second place due to the feedstock requirements, compared to gasification. The biorefinery scenario 1 has the best performance.

**Supplementary Information:**

The online version contains supplementary material available at 10.1007/s11356-024-32241-0.

## Introduction

Energy in all forms is important in developing a country since a reliable and affordable energy supply has been categorized as a pillar of improving living conditions worldwide (Krishnan et al. 2022). However, the unrestricted use of fossil fuels leads to environmental impacts and resource depletion, driven by industrialization and population growth (Saravanan et al. 2023). For this reason, there is a latent need for the energy transition using clean and renewable sources (Amalina et al. 2022). Colombia possesses various energy supply options, with hydroelectric power and fossil fuels as dominant sources due to its geographic location (Ramirez Triana 2012). The use of fossil resources to supply the high energy demand has involved abrupt changes in climate and alterations in ecosystems (Oviedo-Ocaña 2018). In response, Colombia has implemented public policies that focus on the utilization of alternative and renewable energy sources (Departamento Nacional de Planeación de Colombia 2022), (Gobierno Nacional de Colombia 2022). Agricultural and agro-industrial biomass, particularly derived from crops, cereal, and fruit trees, generates substantial waste without any use or application (Ministerio de Agricultura y Desarrollo Rural (Minagricultura) 2021). In the department of Sucre, rice production highlights as one of the most important agricultural activities, resulting in considerable quantities of residues (i.e., straw and husk) (Gobernación de Sucre 2020). Although there are some current uses, the final disposal poses significant challenges and environmental impacts. Because generally burned in open fields for easy disposal, which generates greenhouse gas emissions and particulate matter, causing respiratory diseases (Singh and Patel 2022). On the other hand, the residues generated from the burning of rice residues destabilize the microbiota and the physicochemical characteristics of the soil (Singh et al. 2021; Singh and Patel 2022).

Lignocellulosic biomass has been categorized as a promising solution for sustainable energy generation and value-added compounds production (Krishnan et al. 2022). The availability and physic-chemical properties of these residues are the key characteristics for addressing energy security challenges in developed and developing countries (Saravanan et al. 2023). However, the development and implementation of technologies utilizing renewable resources present ongoing challenges that require further resolution (Wu et al. 2022). The complex composition of biomass and the immaturity of related technologies are the primary obstacles to effective use as an energy source (Maia et al. 2021). In recent years, several research efforts have focused on thermochemical and biotechnological pathways for biomass conversion (Elgarahy et al. 2021). Thermochemical routes, including gasification, pyrolysis, combustion, torrefaction, and hydrothermal liquefaction upgrade biomass into liquid, solid, and gaseous products at high temperatures. The characteristics and yields of these products depend on process configuration and the physicochemical composition of the biomass (moisture content, volatile material, fixed carbon, chemical analysis, and C/H ratio). The resulting products can be converted into electricity, heat, and value-added products (Adeniyi et al. 2023).

Among the thermochemical process, pyrolysis is a well-studied method that involves the breakdown of complex biomass molecules into simple molecules at high temperatures in an anoxic atmosphere (Shafizadeh et al. 2023). This technology has a high potential to produce bioenergy and value-added products (Li et al. 2021b), (Parthasarathy et al. 2022), (Afraz et al. 2024). The pyrolysis process yields bio-oil, biochar, and gases, where the product’s quantity is controlled by process conditions such as residence time, temperature, pressure, heating rate, and particle size (Adeniyi et al. 2023). However, bio-oil presents instability and corrosion due to the high oxygen content and low calorific value when biomass is subjected to a conventional pyrolysis process (Dai et al. 2017) (Li et al. 2021a). For these reasons, different technologies have been studied to carry out bio-oil deoxygenation, in situ or ex situ. The use of catalysts is highlighted, which allow the improvement of the physical and chemical properties of the bio-oil, as well as decrease the oxygen content and total acidity, and increase the calorific value (Sorunmu et al. 2020) (Saravana Sathiya Prabhahar et al. 2020).

A work developed by Li et al. (2021b) studied the fast microwave-catalyzed pyrolysis of rice husk in fixed-bed and fluidized-bed reactors. The authors found that the fluidized-bed reactor increased bio-oil production compared to the fixed-bed reactor of 47.6 to 55.3% by weight of bio-oil. Mohammed et al. (2017) performed a work focused on the co-pyrolysis of rice husk with Naiper grass and sago residues, which resulted in a yield of 34.13, 35.87 and 30% of bio-oil, gases and biochar, respectively. In reference to rice straw, Bhatnagar et al. (2022) performed a slow pyrolysis of rice straw obtaining 38.2, 36.8 and 25% biochar, bio-oil and gases, respectively. Biswas et al. (2022) studied the properties of biochar obtained from rice straw at different temperatures of slow pyrolysis, where thermogravimetric analysis, X-ray diffraction, scanning electron microscopy and Fourier transform infrared spectroscopy had shown that the biochar obtained at 350 and 450 °C presented good thermal stability being the most promising for energy production.

Gasification is another striking thermochemical technology addressed to produce electricity and heat by reacting biomass with an oxidizing agent, such as oxygen, water steam or air. The process consists of three stages, (1) drying, (2) pyrolysis and (3) gasification (Aneke and Wang 2017). The first stage occurs at 105 °C allowing the removal of water in excess in biomass, the second stage takes place between 400 and 500 °C where the volatile pyrolytic components are released to generate bio-oil, bio-char and gases. Finally, the bio-oil and biochar produced in the previous stage are cracked to maximize the generation of synthesis gas in a range of 800 to 1000 °C (Aneke and Wang 2017). The yield and quality of the gasification products depend on several process variables (Verma et al. 2023). Chiang et al. (2016) studied the gasification process of rice straw at different temperatures (*i.e.,* 700, 800 and 900 °C), where low heating values (LHV) of the synthesis gas were 16.5, 18.2 and 15.2 MJ/Nm^3^, and the tar yields were 43, 16.5 and 11 g/kg of biomass, respectively. Pei et al. (2020) found that increasing the temperature from 650 to 800 °C, the energy ratio of syngas increased from 27.2 to 64% and the calorific value was 6.62 MJ/Nm^3^ at 800 °C. For rice husks, Manatura et al. (2017) investigated the air–fuel ratios (ER) that improved syngas yields and energy efficiency.

To address these issues, this research proposes the energy valorization of waste from the rice production chain in Sucre, Colombia. Two thermochemical routes such as pyrolysis and gasification to generate electricity and value-added products are involved. The routes are assessed from techno-economic, energy and environmental perspectives using four biorefinery scenarios to determine its feasibility and performance. These analysis and comparisons contribute to solve those problems associated with the waste disposal and promoting the efficient use of residual biomass in the Department. The novelty of this research is focused on the identification of the biorefinery with the best techno-economic, energetic, and environmental performance in the Colombian context. Economic and environmental analyses were done by using economic metrics and emissions.

## Materials and methods

### Physico-chemical and structural characterization of rice wastes

#### Samples preparation

The rice husk and rice straw were provided by a rice-producing facility placed at San Marcos—Sucre, in the northern region of Colombia. The residues were sun-dried and milled until a particle size of 0.425 mm (ASTM—40 Mesh) (Hames et al. 2008). The physico-chemical characterization (*i.e.,* proximate, elemental, and chemical analysis) of raw materials was carried out in triplicate.

#### Proximal analysis

The proximate analysis involves ash, volatile matter, moisture, and fixed carbon determination. The protocols reported on the (ASTM E17–5—01 2008), (Annual Book of ASTM Standards 2013) and (ASTM E871 – 82 2014) were applied, respectively. Finally, the fixed carbon was estimated as the difference between the ash and volatile matter content on a dry basis. Additionally, the high heating value (HHV) was determined using an IKA C-6000 bomb calorimeter based on (ASTM E711-87 2004). A dried sample of 1.3 g was compressed as a pill and was taken to the bomb, which is equipped with isoperibol adiabatic heating.

#### Elemental analysis

The ultimate analysis allows the quantification of C/H/O/N in a sample. This assay was not carried out in triplicate due to the high precision of the equipment (> 99%). This analysis was carried out using 7 mg of dried sample and the standard (ASTM D591-92 2021) with the EMA 502 Elemental Analyzer CHNS-O.

#### Chemical analysis

The chemical composition of feedstock was determined using the NREL standards (National Renewable Energy Laboratories). The extractives content was quantified using NREL TP-510–42619 (Sluiter et al. 2008). The determination of holocellulose and cellulose, and Klason’s lignin was carried out according to ASTM D1104 and ASTM D1106, respectively (Han and Rowell 1997).

#### Thermogravimetric analysis (TGA)

The TGA was carried out on the thermogravimetric analyzer SDT-650 in an atmosphere rich in N_2_, taking a sample amount of approximately 7 mg. Data were obtained in terms of overall mass loss as a function of temperature, from room temperature up to 950 °C, with four heating rates (β), 5, 10, 20 and 30 °C min^−1^. Subsequently, the data were collected and processed with OriginPro 2022 software. The results were analyzed using three isoconversional methods, which are integral methods based on the Arrhenius equation (Maia and de Morais 2016). The models used in this research are shown in Table 1S of the Supplementary information. These models were Flynn–Wall–Ozawa-Doyle (FWOD), Ozawa-Flynn-Wall (OFW), and Kissinger–Akahira–Sunose (KAS). The activation energy (Ea) was calculated from the slope of the graph relating 1/T to log(β) for FWOD, 1/T to Ln(β) for OFW, and 1/T to Ln(β/T^2^) for KAS. The starting point was data obtained from differential thermogravimetric curves (DTG) where the temperatures are associated with the conversion interval. Finally, the pre-exponential factor (A) was estimated with Vyazovkin’s method (Koga et al. 2023).

#### FTIR spectroscopy

FTIR analysis in transmittance mode was performed on rice husk and rice straw with a Thermoscientific Nicolet magna I5 infrared spectrophotometer. The samples were measured with the 2% KBr pellet technique, using the powdered samples sieved on 40 mesh in a wavelength range between 400 and 4000 cm^−1^. Signals were obtained for the organic and inorganic functional groups present in the samples. The results were processed using OriginPro 2022 software to obtain the infrared spectra, which were subsequently analyzed.

#### Processes description

Rice husk and rice straw were used as raw materials in thermochemical pathways (*i.e.,* pyrolysis and gasification) for the energy generation as main product. The calculation basis for rice straw and rice husk were 1.67 and 1.59 ton/h, respectively. These values correspond to 50 and 10% of the husk and straw produced in the Departament de Sucre for 2021, respectively. The mentioned percentages were selected due to the complex logistics of collection around the straw and the alternative but basic uses of the husk. Additionally, the experimental chemical characterization was the base line to generate the mass and energy balances. Pyrolysis and gasification processes were assessed through four scenarios as shown in Table 1. A detailed description of each thermochemical pathway is presented below.

**Table 1:** Proposed scenarios for pyrolysis and gasification of rice husk and straw

Scenario I	Scenario II
Rice husk pyrolysis + Rice straw pyrolysis	Rice husk gasification + Rice straw gasification
Scenario III	Scenario IV
Rice husk pyrolysis + Rice husk gasification	Rice straw pyrolysis + Rice straw gasification

#### Equipment quotes

**Fig. 1:**
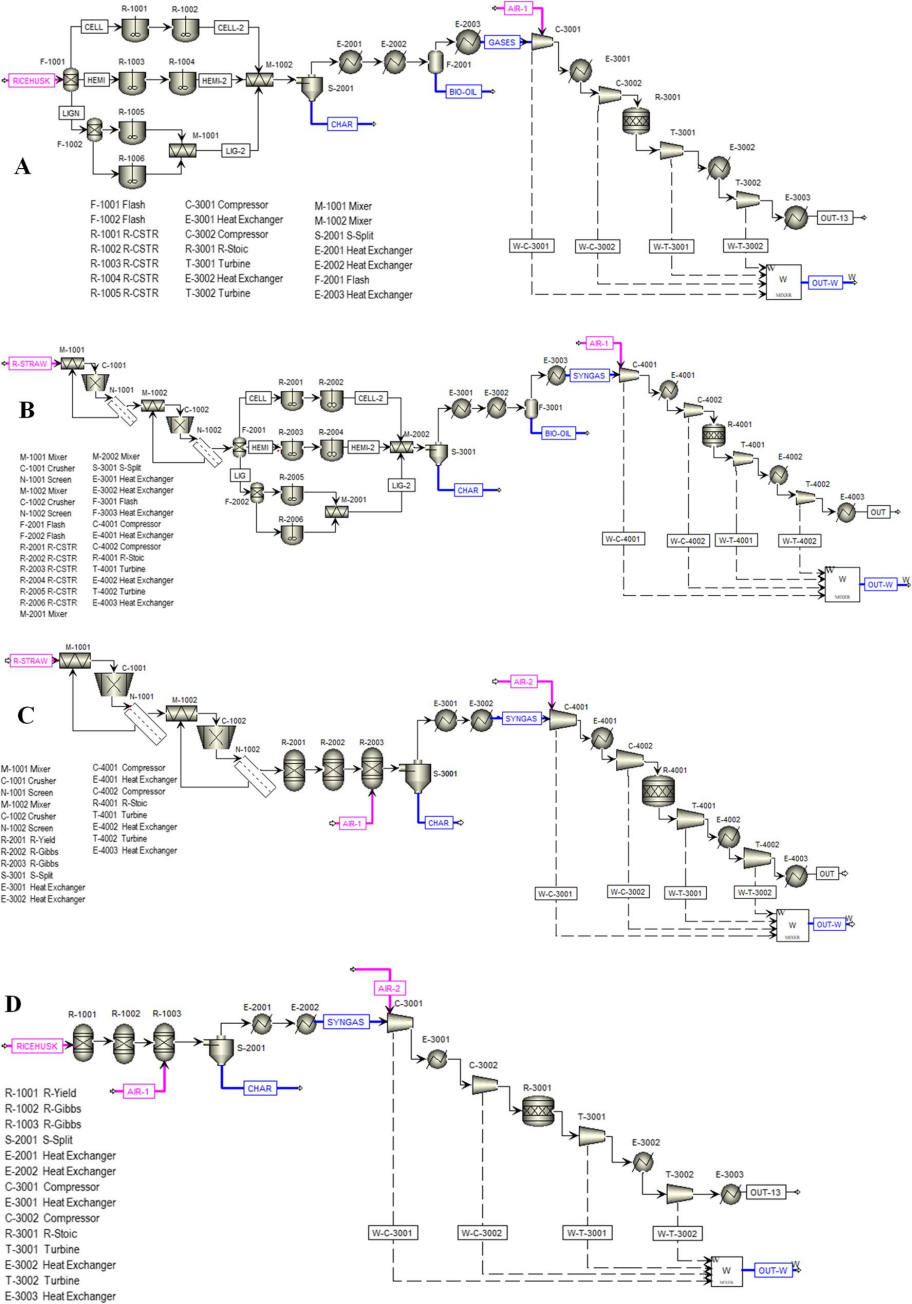
Flowsheet of **A**, **B** pyrolysis of rice husk and straw. **C**, **D** gasification of rice straw and husk, respectively

Four stages were used for the fast pyrolysis process of biomass. Figure 1A and B indicates the flowsheet of pyrolysis process of pyrolysis of rice husk and straw, respectively. First stage considered a particle size reduction applied particularly to rice straw. In this stage, the material was reduced from 25 to 1 cm using two rotary mills. On the contrary, the rice husk did not require particle size reduction before pyrolysis reactions. Then, the second stage involved CSTR reactors that were simulated based on kinetics reported by Humbird et al. (2017). The pyrolysis reactors operated in an inert environment at 500 °C and atmospheric pressure with a residence time of 2 s as reported (Safarian et al. 2022). The third stage corresponded to separation and purification of pyrolysis products. The resulting stream from reaction passed to a cyclone to separate the solid fraction (biochar) from the vapors. These last one was submitted to a condensation through two heat exchangers to separate those molecules that composed the bio-oil. Finally, the following assumptions were taken into account for the simulation process: The bio-char only consists of C and ash, the ash content is inert and the process runs continuously in steady state (Nyambura et al. 2023).

#### Gasification

Four stages were also used for the gasification process of rice residues. Figure 1C and D indicates the flowsheet of gasification rice straw and husk process, respectively. The first stage considered a particle size reduction applied particularly to rice straw. This stage was the same applied to the pyrolysis process. In the second stage, the gasification reactor was simulated following an approach based on the Gibbs free minimization (Sharma 2011), the temperature in the reactor was specified at 580 °C according to the calculation of the adiabatic flame temperature (Rios Escalante et al. 2020) of the biomass as specified in Section 1 of the Supplementary information. Finally, the used air–fuel (ER) ratio was 0.30. The third stage consisted of a cyclone that separates the solid fraction (char and ash) from the gases, the gas stream rich in energy carriers (H_2_, CO and CH_4_) undergoes a cooling process with a pair of heat exchangers. Finally, the fourth stage allowed the simulation of power generation through the synthesis gas combustion which was upgraded in a gas turbine based on the Bryton cycle (Brigljević et al. 2019). The following assumptions were taken into account for the simulation process: The bio-char only consists of C and ash, the ash content is inert, the process runs continuously in steady state and the gaseous compound possess ideal gas behavior (Nyambura et al. 2023).

### Techno-economic and energy analysis

The scenarios were assessed from technical perspective using mass and energy indicators that are shown in Table 2S of the Supplementary information. Product yield, annual production, carbon conversion efficiency (CCE) and cold gas efficiency (CGE) were calculated as mass indicators. Specific energy consumption, overall process efficiency and resource energy efficiency were calculated as energy indicators, considering biochar and syngas as energy vectors (García et al. 2018). The mass and energy balance were the base to determine the indicators. The mass and energy balances for four scenarios mentioned before were calculated via simulation procedures using the software Aspen Plus (Aspen Technology, Inc., USA). Peng Robinson was taken as thermodynamic method. Equipment quotes were used to calculate the costs associated to the processes (CAPEX and OPEX), using as a basis the estimates proposed by Peters and Timmerhaus (1991). This analysis was estimated in US dollars considering the straight-line depreciation method applied to 20 years, using economic parameters of Colombian context, with an interest rate of 13.25% and tax rate of 35%, data reported by the bank of the Republic of Colombia for 8 August 2023 (Colombia 2023). A wage of USD$1.29/h for operators was considered, according to the current legal minimum wage and market representative rate (TMR). A plant operation time of 24 h with 3 shifts of 8 h was assumed. Economic margin was used to quantify the economic performance of assessed scenarios, as well as, CAPEX (based on fixed capital costs of equipment), and OPEX (calculated as the sum of costs of raw materials, utilities, maintenance, labor, fixed and general costs and overhead), and the general profits from the product were discussed (García-Velásquez et al. 2018). As calculation basis for rice husk and rice straw was considered the 50% and 10% of the total production of each waste generated in Sucre—Colombia, respectively, because they have other uses in the region. On the other hand, the price stipulated for the raw materials and utilities was taken from the sale price in the region. Table 2S of the Supplementary information shows prices of raw materials, utilities and products of pyrolysis and gasification processes.

### Environmental assessment

The environmental assessment of the processes studied in this research was done only to assess the Global Warming Potential (GWP) of the (IPCC 2007). This environmental category was selected based on the type of processes analyzed, which generated large amounts of greenhouse gases. The GPW was studied in different time periods (20, 100 and 500) years, evaluating the long-term impacts of the gas streams emitted to the air. Generally, the GWP is expressed in kgCO_2_eq/kg and the equivalence to kgCO_2_ of other considered greenhouse gases are shown in Table 3S of the Supplementary information.

## Results and discussion

### Physicochemical and structural characterization

The physicochemical analysis of rice husk and straw samples provide an overview of the applicable technologies, in this case pyrolysis and gasification. The results obtained in this research and those reported by other authors are shown in Table 2. Rice residues have a moisture content of 8.31% and 9.50% for rice husk and straw, respectively. These data are like values reported in other papers. Moisture content in biomass is one of the crucial factors in thermochemical applications since high moisture content can affect the energy efficiency of the processes and the products composition (i.e., bio-oil and syngas) (Bisht and Thakur 2019).

**Table 2:** Physicochemical characterization of rice husk and rice straw

	This work	Other authors	
Rice husk			
Component	**%**	(Chen et al. 2021)^b^	(Titiloye et al. 2013)^b^
Moisture (%)	8.31 ( ±) 0.17	7.73	8.59
Proximal analysis			
Ash (%)	21.67 ( ±) 0.04	12.57	24.71
Volatile matter (%)	67.21 ( ±) 0.27	64.20	58.22
Fixed carbon (%)^a^	11.11 ( ±) 0.14	15.50	8.48
Elemental analysis			
Carbon (%)	36.79	38.62	34.9
Hydrogen (%)	4.74	5.67	5.15
Nitrogen (%)	0.75	0.48	0.31
Sulfur (%)	< 0.01	NR	0.64
Oxygen (%)^a^	57.7105	41.38	59
HHV (MJ/kg)	14.75 ( ±) 0.09	15.39	NR
Chemical analysis			
Cellulose (%)	34.62 ( ±) 0.84	38.57	37.34
Hemicellulose (%)	11.01 ( ±) 0.33	21.30	10.07
Lignin (%)	46.63 ( ±) 4.51	21.10	41.08
Extractives (%)	7.72 ( ±) 0.68	NR	11.51
Rice straw			
Component	**%**	(Chen et al. 2021)^b^	(Wei et al. 2023)^b^
Moisture (%)	**9.50 ( ±) 0.12**	**8.08**	**5.64**
Proximal analysis			
Ash (%)	16.93 ( ±) 0.01	8.79	19.22
Volatile matter (%)	70.01 ( ±) 0.59	69.99	74.4
Fixed carbon (%)^a^	13.04 ( ±) 0.53	13.40	6.38
Elemental analysis			
Carbon (%)	37.43	39.61	42.99
Hydrogen (%)	5.18	5.38	5.18
Nitrogen (%)	0.94	1.21	0.84
Sulfur (%)	< 0.01	NR	0.16
Oxygen (%)^a^	56.43	43.80	31.61
HHV (MJ/kg)	14.47 ( ±) 0.16	14.21	NR
Chemical analysis			
Extractives (%)	14.30 ( ±) 0.86	NR	NR
Cellulose (%)	38.27 ( ±) 0.86	37.65	47.72
Hemicellulose (%)	9.24 ( ±) 1.44	31.82	11.23
Lignin (%)	38.18 ( ±) 2.13	6.18	33.32

^a^ Calculated by difference

^b^ Dry basis

*HHV*, high heating value

Likewise, the ash, volatile material, and fixed carbon contents obtained for both samples are like the reported data. The ash content can be considered to define the amount of minerals present in the biomass which can act not only as catalysts during the secondary reactions of the process (Duong et al. 2019), but also cause accumulation in the reactor and clinker (Bisht and Thakur 2019). Fixed carbon can be used to estimate the minimum amount of carbon from photosynthetic carbon fixation in the form of CO_2_ in biomass and carbonaceous products formed during the pyrolysis process (Basu 2013). On the other hand, the volatile material estimates the amount of condensable (bio-oil) and non-condensable (gases) products during pyrolysis (Basu 2013). The same happens in gasification, the volatile material present in the biomass is volatilized from the solid in the pyrolysis stage. And then the biochar formed during this stage is thermally degraded in gasification to become part of syngas or tar (Bisht and Thakur 2019).

The values obtained in the elemental analysis for both biomasses are similar to the data reported in the literature (Chen et al. 2021), (Titiloye et al. 2013). These results may show some characteristics of the fuel under study, as the O/C and H/C ratios indicate the quality of the fuel in terms of calorific value. In this sense, the results of the O/C ratios are 1.17 and 1.13 for rice husk and rice straw, respectively. The H/C ratio is 1.52 for husk and 1.65 for straw, which are likely reported for biomass thermochemical conversion (i.e., according to the Van Krevelen) (Basu 2013). These results can be correlated with the higher calorific value of biomass14.75 MJ/kg and 14.47 MJ/kg for husk and straw, respectively. H/C and O/C ratios above 0.9 reduce the calorific value of the material to around 15 MJ/kg (Basu 2013). On the other hand, biomass has a low nitrogen content, which reduces the production of the NOx (Bisht and Thakur 2019).

The cellulose and hemicellulose contents obtained are like those referenced in Table 2, unlike lignin that differ from the studies reported by other authors. This can be attributed to the rice variety FEDEARROZ 2000, crop management and environmental conditions (Aristizábal-Marulanda et al. 2021). In thermochemical processes such as pyrolysis, the yield and composition of bio-oil can be influenced by hemicellulose and cellulose content. Lignin, together with ash, promotes biochar production (Duong et al. 2019). The studies cited above do not report data on extractives.

**Fig. 2:**
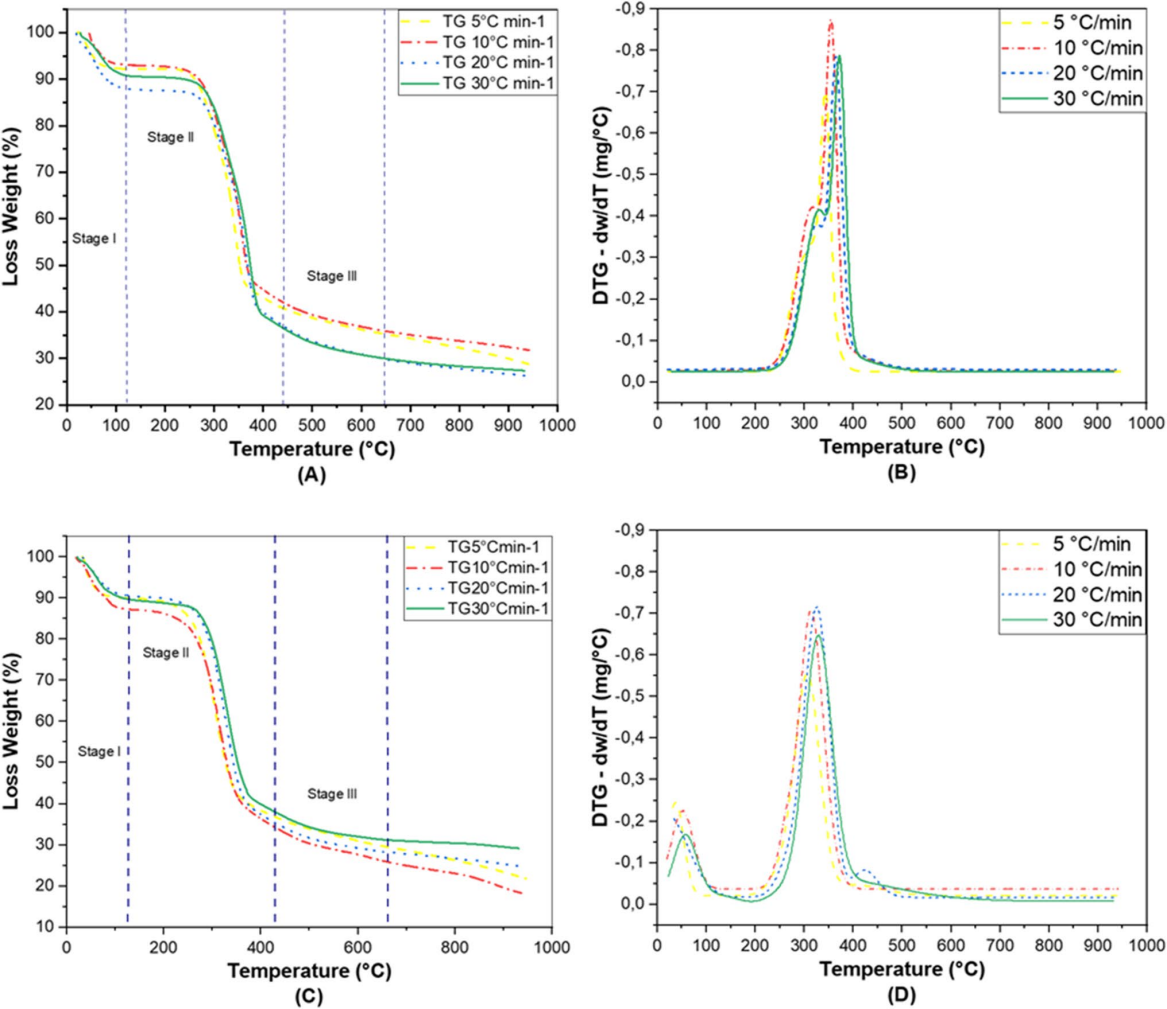
**A** and **B** TGA y DTG of rice husk. **C** and **D** TGA y DTG of rice straw

TGA of the rice samples was carried out at different heating rates. Figure 2 shows the biomass decomposition process using thermogravimetric (TG) curves where the behavior of the % mass loss as a function of temperature and DTG curves representing the mass loss rate as a function of temperature for rice husk and straw with 4 heating rates 5, 10, 20 and 30 °C/min are observed. In general, this figure shows a similar behavior in the biomasses under study. However, it can be observed that the samples react differently at the specified heating rates. This allows us to understand that the maximum temperatures in each of the stages do depend on the heating rate, while the mass loss does not depend on this condition (Chen et al. 2021), (Xu et al. 2022). In Fig. 2A and C it can be observed that in stage l, from room temperature to approximately 110 °C, there is a mass loss of about 10% attributed to the volatilization of moisture, which is confirmed by the peaks shown in the DTG curves located on the left side of Fig. 2B and D for husk and straw, respectively.

Likewise, stage II (active pyrolysis) can be observed in Fig. 2A and C, where a higher mass loss of about 60% is observed for both biomasses at all heating rates. This degradation ranges from 110 to 450 °C and is due to the volatilization of hemicellulose components and part of the cellulose. The same figures show stage III (passive pyrolysis), which has a lower mass loss than stage III, approximately 15–20%. This is due to the volatilization of lignin, which occurs between 450 and 660 °C. Finally, at 660 °C and above, the TG and DTG curves for both biomasses tend to be flat due to the formation of biochar and ash (Chen et al. 2021), (Gajera et al. 2020), (Narnaware and Panwar 2022).

Figure 2B and D corresponds to the DTG diagrams of rice husk and rice straw, respectively. These figures are useful for determining the ranges of decomposition temperatures of biomass structural components such as hemicellulose, cellulose and lignin. Several authors have reported that decomposition temperatures are approximately in the ranges of 220–315 °C, 300–450 °C and 392–750 °C, respectively (Gajera et al. 2020), (Narnaware and Panwar 2022), (Di Blasi 2008), (Açıkalın 2021), (Wang et al. 2017). Thus, Fig. 2B for the different heating rates shows that the general degradation occurs between 245.14 °C where the shoulder starts and thus the volatilization of hemicellulose goes up to 345.88 °C with a range of mass loss between 22.94 and 35.66%, showing it to be the component with the least thermal stability. The same behavior is shown in Fig. 2D, but with temperature ranges that go from 245.35 °C (where the shoulder starts, which is less pronounced compared to that observed in Fig. 2B, due to the low percentage of hemicellulose) up to 296.76 °C, with mass loss ranges between 19.49 and 23.69% (Chen et al. 2021). Figure 2B shows the characteristic sharp peak of cellulose decomposition in the temperature range from 305.53 to 387.35 °C, with mass losses between 50.21 and 54.26%. For Fig. 2D, decomposition occurs between 272.94 and 368.03 °C, with mass losses between 52.61 and 56.25% for the different heating rates. Finally, the representative curve for lignin is between 357.53 and 442.32 °C for Fig. 2B with mass losses between 55.18 and 58.05%. For Fig. 2D the temperature range is 332.85 to 555.91 °C with mass losses between 65.01 and 69.50%.

Due to the complexity of the biomass structure, it is necessary to use alternatives for the analysis of behavior in the degradation process and thermal stability (Koga et al. 2023). This can be solved by studying biomass kinetics with isoconversional methods. The FWOD, OFW and KAS methods are also called model-free integral methods, have an advantage over differential models due to the minimization of experimental noise (Choudhary et al. 2022). In addition to being identified by, not considering the reactions given during the decomposition process, allowing a one-step approximation of the reaction to be obtained, provided that the activation energy calculated by these methods does not vary significantly with respect to the mass loss rate (Chen et al. 2021), (Narnaware and Panwar 2022).

**Fig. 3:**
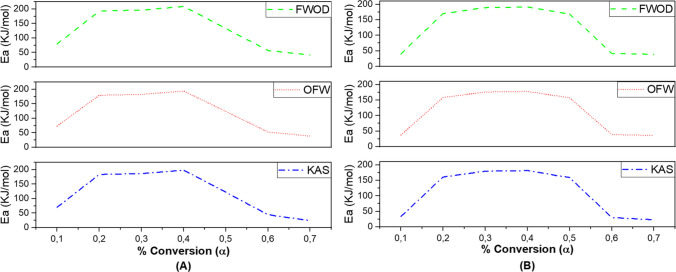
Variation of activation energy Vs conversion factor **A** rice husk and **B** rice straw

The Ea and A are shown in Table 3. Estimation of kinetic parameters by various methods generates confidence in obtaining results. Table 3 shows the results obtained using the FWOD, OFW and KAS models for Ea and A. Is observed that the Ea experienced a gradual increase in the conversion zone from 0.2 to 0.5 for husk and straw rice, showing a very similar behavior. This zone can be associated with the active pyrolysis zone (Narnaware and Panwar 2022). Since this is where the largest kinetic reactions take place, corresponding to zone II in Fig. 2A and B. Figure 3 shows the behavior of the Ea with respect to the conversion value. The first stage shows the lowest Ea values, due to the release of the volatile components with lower molecular weight of the biomass such as moisture (Fernandes et al. 2013). This influences the requirement of a lower kinetic energy to initiate the reaction of breaking chemical bonds between molecules (Fernandes et al. 2013).

**Table 3:** Kinetic parameters of the FWOD, OFW and KAS models

Conversion									
	KAS			OFW			FWOD		
	Ea	A	*R*^2^	Ea	A	*R*^2^	Ea	A	*R*^2^
Rice husk									
0.1	70.442	5.07E^06^	0.99	73.5	1.12E^07^	0.99	79.311	2.04E^07^	0.96
0.2	183.59	4.46E^16^	0.95	179.57	1.84E^16^	0.96	193.11	3.61E^17^	0.99
0.3	186.5	1.34E^16^	0.87	182.69	6.01E^15^	0.87	196.45	1.08E^17^	0.87
0.4	198.513	2.73E^16^	0.98	194.28	1.16E^16^	0.98	208.92	2.22E^17^	0.99
0.5	122.37	3.13E^09^	0.88	123.84	4.19E^09^	0.89	133.16	2.96E^10^	0.91
0.6	44.683	1.53E^02^	0.92	52.887	9.98E^02^	0.93	56.87	1.44E^03^	0.95
Rice straw									
0.1	33.65	4.87E^03^	0.98	37.796	1.86E^04^	0.96	40.647	4.62E^04^	0.95
0.2	160.884	4.13E^14^	0.91	158.258	3.96E^14^	0.92	170.18	3.14E^15^	0.92
0.3	179.707	1.13E^15^	0.87	176.066	5.14E^15^	0.88	189.32	4.27E^16^	0.88
0.4	181.428	5.62E^15^	0.91	177.912	2.67E^15^	0.92	191.31	2.37E^16^	0.92
0.5	158.756	1.72E^13^	0.91	157.172	1.25E^13^	0.92	169.00	1.11E^14^	0.92
0.6	30.123	1.80E^01^	0.99	39.028	9.17E^01^	0.99	41.968	1.54E^02^	0.99

Ea: Activation energy (kJ/mol)

A: Exponential factor (s^−1^)

*R*^2^: Coefficient of correlation

The isoconversional models of FWOD, OFW and KAS present global kinetic parameters close to each other and with a dispersion data correlation (*R*^2^) higher than 0.9, which allows then estimation to be reliable**.** Figure 3A and B represents the variation of Ea with conversion factor. The three methods show a similar trend of the Ea curve with increasing conversion factor. The average Ea for rice husk was 134.35 kJ/mol and rice straw was 124.09 kJ/mol.

The estimated values are similar to data reported by Tian et al. (2021) where the Ea for rice husk ranges between 140 and 200 kJ/mol up to 318 °C the decomposition was attributed to hemicellulose and for cellulose around 200 kJ/mol with the FWO and KAS methods. Other investigations show that the activation energy for rice husk by OFW and KAS methods are in the ranges of 149–241 kJ/mol with an A between 2.13*10^12^ -5.79*10^15^ s^−1^ and an Ea of 151-251 kJ/mol and 3.43*10^12^—6.80*10^15^ s^−1^, respectively (Choudhary et al. 2022), (Kumar et al. 2020). On the other hand, for rice straw, the activation energy has been reported to be in the range of 117.29 and 208.36 kJ/mol (Chen et al. 2021). Other studies have also reported kinetic parameters with Ea of 126.31 kJ/mol and A of 9.57*10^9^ s^−1^ for hemicellulose. For cellulose, a kinetic energy of 223.32 kJ/mol and 3.5*10^12^ s^−1^ as A (Zhu and Zhong 2020) (Titiloye et al. 2013).

**Fig. 4:**
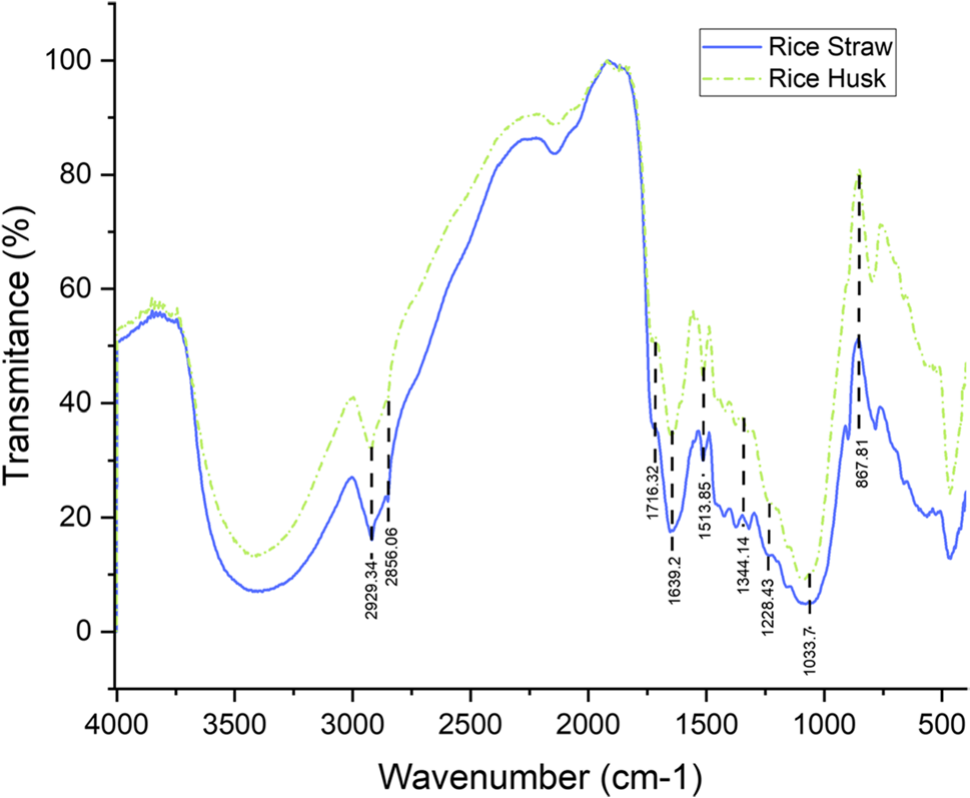
FTIR results of rice husk and rice straw

FTIR analysis provides information about the functional groups present in rice husk and straw samples. Initially, it is shown that rice husk and rice straw behave under the same trend. However, they differ in the intensity of the signals indicating that the husk contains more specific molecules in each signal. The spectrum also shows that the main components of biomass, in this case hemicellulose, cellulose and lignin, are made up of esters, alcohols, carboxylic acids and aliphatic and aromatic compounds (Zafeiropoulos et al. 2003). Figure 4 and Table 4S of the Supplementary information show the infrared spectra and band assignments for both residues.

Initially, the two samples indicate the absorption band between 3600 and 3000 cm^−1^ which corresponds to the stretching vibration of the hydroxyl group (O–H) that can belong to water (moisture in the sample), alcohols, phenols, carbohydrates and carboxylic acids (Kumar et al. 2020), (Le Troedec et al. 2008), (Thakur et al. 2018). Following the Sp^3^ stretches of the C-H bonds belonging to polysaccharides and aliphatic and aromatic compounds (Yang et al. 2007), (Le Troedec et al. 2008). The signals at 2929.34 and 2856.06 cm^−1^ are assigned to the asymmetric and symmetric vibrations of the C-H bonds of the methyl, methylene and alkyl groups belonging to cellulose and hemicellulose (El-Hendawy 2006). On the other hand, the signal located at 1716.32 cm^−1^ belongs to the stretching vibration of the acetyl C = O group of the hemicellulose structure (Choudhary et al. 2022), (Le Troedec et al. 2008). The C–C associated vibration is observed at 1639.2 cm^−1^ for hemicellulose and lignin (Kumar et al. 2020). The signal shown at 1513.85 cm^−1^ is attributed to the vibration of the phenylpropane skeleton for the C = C bonds and at 1344.14 cm^−1^ for C-H of the phenolic groups, both signals representative of lignin (Kumar et al. 2020), (Alriols et al. 2009). As these are silica-rich samples, the signal at 1228.43 cm^−1^ from the vibration of the O-Si–O bonds can be observed (Choudhary et al. 2022), (Kumar et al. 2020). Finally, between 1200 and 1000 cm^−1^ signals of C-O bonds from hemicellulose and C-H bonds from cellulose and lignin can be observed, additionally the signal of amorphous cellulose is observed at 867.81 cm^−1^ (Choudhary et al. 2022).

### Techno-economic results

Table 4 shows the results obtained from the mass balance of each of the raw materials. It is observed that the flows of husk and straw are initially comparable, which allows to easily observe which scenario has the best performance. In terms of yield, rice straw is the raw material that shows the best performance to produce bio-oil by pyrolysis. On the other hand, rice husk generates more electricity than straw by using the synthesis gas obtained from gasification.

**Table 4:** Inputs and outputs streams of pyrolysis and gasification for rice husk and straw. Economic results for the four scenarios

Input	Rice straw pyrolysis	Rice husk pyrolysis		Rice husk gasification	Rice straw gasification		
	Flow (Ton/h or KW/h)	Flow (Ton/h or KW/h)		Flow (Ton/h or KW/h)	Flow (Ton/h or KW/h)		
Biomass	1.67	1.59		1.59	1.67		
Air	-	-		1.62	1.84		
Output							
Electricity	-	-		792.55	645.22		
Bio-oil	1.19	1.05		-	-		
Bio-char	0.36	0.41		0.38	0.39		
Item	Scenario 1			Scenario 2			
	Rice Straw Pyrolysis	Rice Husk Pyrolysis	Total	Rice Straw Gasification	Rice Husk Gasification		Total
	Cost (mUSD/year)			Cost (mUSD/year)			
Raw Materials	0.666	0.634	1.300	0.666	0.634		1.300
Utilities	0.401	0.300	0.701	0.428	0.381		0.809
Maintenance	0.018	0.018	0.036	0.024	0.022		0.046
Labor	0.062	0.062	0.123	0.027	0.041		0.068
Fixed & General	0.022	0.022	0.044	0.019	0.020		0.040
Plant Overhead	0.042	0.042	0.084	0.027	0.033		0.060
Laboratory charges	0.009	0.009	0.019	0.004	0.006		0.010
Insurance and taxes	0.005	0.005	0.010	0.007	0.006		0.013
Administrative cost	0.011	0.011	0.021	0.007	0.008		0.015
Capital Depreciation	0.041	0.041	0.082	0.055	0.050		0.105
Total	**1.277**	**1.144**	**2.421**	**1.266**	**1.201**		**2.467**
Products	**Cost (mUSD/year)**		**Total**	**Cost (mUSD/year)**			**Total**
Electricity	-	-	-	0.830	1.019		1.849
Bio-oil	1.313	1.163	2.551	-	-		-
Bio-char	0.155	0.177	0.332	0.246	0.161		0.621
Total	**1.468**	**1.340**	**2.808**	**1.076**	**1.180**		**2.256**
% Economic Margin	**12.999**	**14.585**	**13.755**	**-17.674**	**-1.705**		**-9.317**
Item	Scenario 3			Scenario 4			
	Rice Husk Pyrolysis	Rice Husk Gasification	Total	Rice Straw Gasification	Rice Straw Pyrolysis	Total	
	Cost (mUSD/year)			Cost (mUSD/year)			
Raw Materials	0.634	0.634	1.269	0.666	0.666	1.332	
Utilities	0.300	0.381	0.681	0.428	0.401	0.830	
Maintenance	0.018	0.022	0.039	0.024	0.018	0.042	
Labor	0.062	0.041	0.102	0.027	0.062	0.089	
Fixed & General	0.022	0.020	0.042	0.019	0.022	0.041	
Plant Overhead	0.042	0.033	0.075	0.027	0.042	0.069	
Laboratory charges	0.009	0.006	0.016	0.004	0.009	0.014	
Insurance and taxes	0.005	0.006	0.011	0.007	0.005	0.012	
Administrative cost	0.011	0.008	0.019	0.007	0.011	0.017	
Capital Depreciation	0.041	0.050	0.091	0.055	0.041	0.097	
Total	**1.144**	**1.201**	**2.345**	**1.266**	**1.277**	**2.543**	
Products	Cost (mUSD/year)		Total	Cost (mUSD/year)		Total	
Electricity	-	1.019	1.019	0.830	-	0.830	
Bio-oil	1.163	-	1.163	-	1.313	1.364	
Bio-char	0.177	0.161	0.338	0.246	0.155	0.401	
Total	**1.340**	**1.180**	**2.520**	**1.076**	**1.468**	**2.544**	
% Economic Margin	**14.585**	** − 1.705**	**6.951**	** − 17.674**	**12.999**	**0.028**	

In this work, the cost of nitrogen for pyrolysis was not taken into account, because from flows 100 kg/h biomass produce quantities of gases that can be recirculated and act as biomass fluidizer (Fadhilah et al. 2023), (Dutta et al. 2016).

The cost obtained for the technologies applied to biomass in this study are shown in Table 4 in millions of dollars (mUSD). The operating costs of the proposed scenarios include the cost of raw materials, utilities, maintenance, labor, fixed and general charges and depreciation**.** The cost of raw material is the one that has the highest participation in operating cost, being 0.666 mUSD and 0.634 mUSD for straw and husk, respectively. This price was supplied by the rice millers of the Department of Sucre, because of being marketed for poultry, bovine, and swine transport bedding. However, this market does not have the consumption capacity of the total raw material generated (Osorio Aguirre 2019). Secondly, there are the service fluid required for the process. The other operating costs were estimated as reported by Peters and Timmerhaus (1991).

Table 4 also shows the economic margin of the 4 scenarios and of the individual feedstocks. As for the individual feedstocks, it can be seen that gasification for both feedstocks has a negative economic margin, while for pyrolysis both feedstocks are promising to be exploitable by this route. On the other hand, scenario 1 is postulated as the most promising for the utilization of the raw materials. Showing an economic margin of 13.76%, because bio-oil has a high yield and marketing price compared to the price of bio-char. Bio-oil can be used for transformation into high value molecules (Dutta et al. 2016), while bio-char can be used for heat generation through combustion or in bioremediation (Rojas 2020), (Jaider et al. 2009).

Scenario 2 shows an economic margin of − 9.32%, the production and sale of electrical energy and biochar from both raw materials through gasification is not enough for the scenario to be economically prefeasible. This is due to the physicochemical composition in particular in the high oxygen content, low hydrogen content and high H/C and O/C ratios compared to mineral coal (Basu 2013).

Scenarios 3 and 4 show an economic margin of 6.95% and 0.03%, respectively. A difference is observed between the two scenarios; this difference can be attributed to the particle size reduction pretreatment that must be performed on the rice straw, which contributes to the maintenance and equipment costs of the process. In addition, rice straw in gasification generates a lower amount of energy compared to rice husk.

**Fig. 5:**
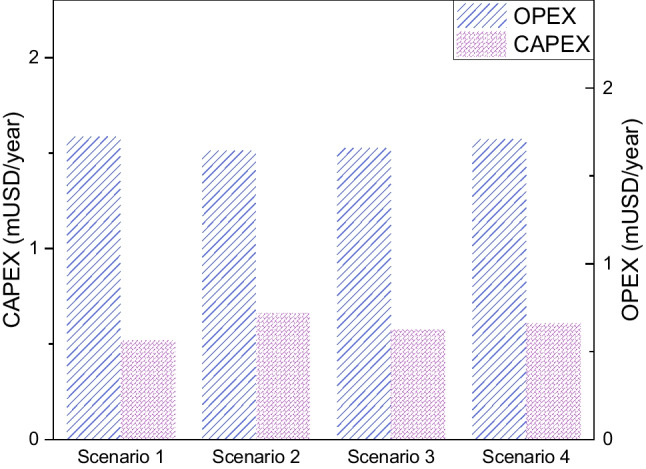
CAPEX and OPEX of the proposed scenarios

On the other hand, Fig. 5 shows the distribution of CAPEX referring to capital costs (equipment costs) and OPEX referring to operating costs (raw material costs, maintenance, labor, fixed and general charges) for each of the proposed scenarios. The OPEX for the 4 scenarios are slightly different from each other, being (1.59, 1.51, 1.53, 1.57) mUSD/year for scenarios 1, 2, 3 and 4, respectively. However, for the CAPEX capital costs, there is a difference between the proposed scenarios. Thus, the scenario that obtained the highest capital cost was scenario 2 with 0.66 mUSD/year, which is due to the acquisition of the combustion chambers for the synthesis gas obtained from gasification. In second place is scenario 4 with a CAPEX of 0.61 mUSD/year, which is influenced by the acquisition of the mill for the pretreatment of rice straw. Finally, scenarios 1 and 3 have a CAPEX of 0.52 y 0.57 mUSD/year, respectively.

The technical analysis, mass and energy indicators are shown in Table 5 Error! Reference source not found.**.** For the yield, it is necessary to clarify that in this work the gases from the pyrolysis process for both feedstocks are not used for electricity production due to their low calorific value (Roda 2012). However, these gases can be recirculated to the reactor and used for biomass drying (Fadhilah et al. 2023), (Dutta et al. 2016).

**Table 5:** Technical analysis, mass, and energy indicators

Item	Scenario 1	Scenario 2	Scenario 3	Scenario 4
Product yield				
* Electricity (kWh/ton)*	-	40.981	49.871	38.666
* Bio-oil (kg/ton)*	68.610		66.041	71.056
* Bio-char (kg/ton)*	23.863	23.720	24.963	22.676
Annual production				
* Electricity (MWh/year)*	-	12,478.826	6981.555	5497.270
* Bio-oil (ton/year)*	19,044.782	-	9127.671	10,492.66
* Bio-char (ton/year)*	6623.985	8644.886	7255.376	7878.610
Carbon conversion efficiency CCE				
* Syngas (%)*	-	63.934	63.546	64.294
* Bio-oil (%)*	19.484	-	35.071	36.184
* Bio-char (%)*	2.271	17.562	11.026	8.885
Lower heating value LHV				
* Syngas (MJ/Nm*^*3*^*)*	-	5.481	5.617	5.345
Cold gas efficiency CGE				
* Syngas (%)*	-	58.823	58.353	59.281
Energy indicator				
* Specific energy consumption (kW/kg biomass) SEC*	131.615	81.247	141.062	157.626
* Overall energy efficiency (%)*	49.772	70.610	57.829	62.691
* Resource energy efficiency (%)*	54.318	78.816	63.429	69.621

In Table 5, it can be seen that the performances are close for all products. Regarding electricity performance, scenario 3 is postulated with the best performance, continuing with 2 and 4 (49.87, 40.98 and 38.67) kWh/ton respectively, defining rice husk as the raw material with the best characteristics to produce electricity.

On the other hand, rice straw has the best performance to produce bio-oil from pyrolysis. As can be seen, scenario 4 has the best performance with 71.06%, continuing with scenario 1 (68.61%) and scenario 3 (66.04%). Finally, the biochar is obtained from all the processing lines and yield is very close to each other with 24.96% for scenario 3, 23.86% for scenario 1, 23.73% for scenario 2 and finally 22.68% for scenario 4.

The carbon conversion efficiency (CCE) relates the amount of carbon present in the biomass and the amount converted into gasification and pyrolysis products. It can be observed that in scenario 1 the carbon is distributed in a higher percentage in the bio-oil with 19.48% and in the bio-char with a CCE of 2.27%. For scenario 2, the carbon is in greater proportion in the synthesis gas with 63.93% and in the biochar in smaller proportion with 17.56%. Finally, for scenarios 3 and 4 the percentages of carbon distribution are similar to each other with a percentage distribution of 63.55 and 64.29% for electricity, 35.05 and 36.18% for bio-oil and 11.03 and 8.89% for biochar, respectively.

The lower heating value (LHV) and the cold gas efficiency (CGE) determine the quality of the gas obtained by gasification and the efficiency of the process. In this sense, in the proposed scenarios the LHV of the synthesis gases do not differ between them for each of the scenarios and are similar to those reported for rice husk (5.85 and 5.1) MJ/Nm^3^ (Yoon et al. 2012), (Tuan et al. 2022). For the CGE, we obtained results of approximately 58.8% for scenarios 2, 3 and 4, which depends on the air–fuel ratio (ER) and is within the range reported by Yoon et al. (2012) being between 50 and 70%.

For the energy analysis, the specific energy consumption (SEC) was estimated, which involves the energy of each line with the total energy consumption of the process, as shown in Table 5. The specific energy consumption lower is for scenario 2 81.25 kW/kg biomass compared to the other scenarios because the gasification reactor is autothermic and energy consumption is due to the equipment attached in the gasification plant. Also, it can be observed that scenario 4 obtained an SEC 157.63 kW/kg biomass. The highest of the scenarios studied because rice straw pyrolysis requires energy for the reactor and decrease in particle size, which lead to an increase in the specific energy consumption. Scenario 2 is followed by scenario 1 and 3 with a SEC of 131.62 and 141.06 kW/kg biomass, respectively, that they do not differ much between them.

The overall energy efficiency was also estimated, which was calculated as the ratio between the energy released by the combustion of the vectors and the overall energy input of the process. Where evident that scenario 2 with 70.61% is the performance since involves the gasification of the 2 raw materials, gasification being a process largely designed to produce energy carriers, for this reason this scenario has a better performance. On the other hand, the lowest performance is presented by scenario 1 49.77%, this is due to the fact that fast pyrolysis is generally used to obtain bio-oil, so the energy vectors do not have a good performance, emissions, which is reflected in a lower overall energy efficiency. Finally, scenarios 3 and 4 have similar overall energy efficiencies (63.43 and 69.62%), respectively, since both scenarios include the gasification of one of the raw materials, which increases energy efficiency.

Finally, the resource energy efficiency of the ratio between the energy content of the products and raw materials. Thus, this indicator postulates scenario 2 as having the best performance with 78.82%, since in this scenario both products obtained from gasification were destined for energy production by combustion. Also observed that scenario 1 is the one with the lowest performance with 54.32%; this is due to the fact that in the pyrolysis of the raw materials, only bio-char is used as an energy vector. Finally, with a slight difference between them, scenarios 3 and 4 are positioned with 63.43% and 69.62%, respectively, where one of the raw materials is gasified in each scenario.

### Environment assessment

**Fig. 6:**
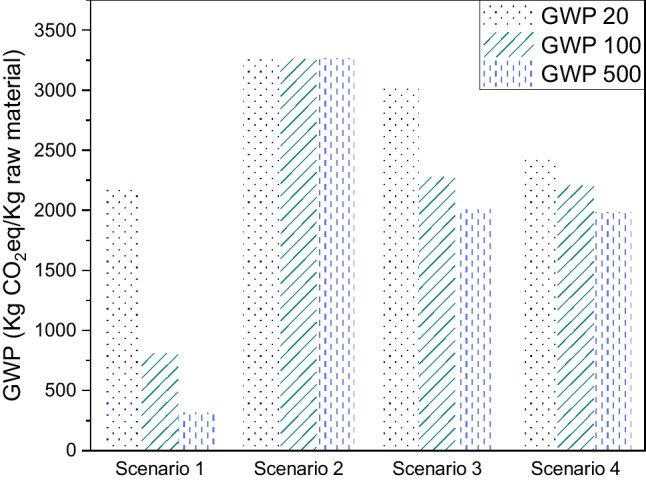
GWP for the proposed scenarios

Figure 6 shows the kgCO_2_eq/kg of raw material for each of the proposed scenarios at 20, 100 and 500 years. The figure postulates scenario 2 as the one that emits the most CO_2_ to the environment over time with 3266.46 kgCO_2_eq/kg of raw material; this scenario proposes gasification and electricity generation from both feedstocks. Therefore, during the combustion of syngas, the most abundant by-product is CO_2_. However, the CO_2_ produced from the combustion of syngas decreases approximately 90% GWP (Akbarian et al. 2022) compared to the direct incineration of biomass. On the other hand, scenario 1 generates fewer emissions since the pyrolysis of both raw materials does not generate high flows of greenhouse gases. Finally, it is observed that GWP for scenarios 1, 3 and 4 decrease with increasing years because greenhouse gases such as methane are treated and removed by natural mechanisms (Catrileo 2008).

Nowadays, the integral use of all by-products obtained from the processing lines has become an important pillar for the efficient management of waste (Moayedi et al. 2019). Thus, the ash in rice residues represents between 15 and 20% and if deposited freely on the soil can cause alterations in the ecosystem due to the inorganic load present, in particular the silicon oxide that makes up 99% of these ashes (Nguyen et al. 2022). However, the presence of silica provides pozzolanic characteristics, which has allowed it to be studied as a cement additive for civil engineering applications, increasing the strength and durability of the material (Moayedi et al. 2019), in addition to being used as raw materials for the synthesis of ceramic materials (Wahab et al. 2020). On the other hand, other research has shown the applicability of rice husk ash as a sorbent in the extraction of antibiotics in solid phase (Grefa et al. 2023). Also, the solid fraction of the pyrolysis process has been studied as a catalyst due to high silica content in transesterification and esterification reactions to produce biodiesel from vegetable oil residues (Li et al. 2014).

## Conclusions

In this research, the techno-economic, energetic, and environmental feasibility of four proposed biorefinery scenarios based on the pyrolysis and gasification of rice husk and straw was analyzed. The results showed that the gasification analyzed as an individual process does not promise to be economically feasible for the studied feedstocks, since the energy generated from syngas and bio-char is not sufficient to cover the process demand and generate profits. On the other hand, the proposed pyrolysis for obtaining bio-oil as a value-added product and bio-char for heat generation shows a positive economic margin. In this way, biorefinery scenario 1, has the best performance. For this scenario, the economic margin and GWP were of 13.76% and 2170.93 kgCO_2_eq/kg for 10 years, respectively. The transformation routes that involved the rice husk as raw material have an advantage over those that used rice straw, due to the particle size and moisture content. These factors influence the capital cost and therefore, the operational cost associated with maintenance and utilities. Finally, the development of this research work demonstrates that the use of biomass from the rice crop and agroindustry is an alternative to reduce the dependency on fossil fuels and promotes a decentralized, efficient, and sustainable energy system.

## Supplementary Information

Below is the link to the electronic supplementary material.

Supplementary file1 (DOCX 114 KB)

## Data Availability

The authors declare that the data supporting the findings of this study are available within the paper and its Supplementary Information files. Should any raw data files be needed in another format they are available from the corresponding author upon reasonable request.
