# Supplementary material for: Assessment and comparison of thermochemical pathways for the rice residues valorization: pyrolysis and gasification
Source: Environ Sci Pollut Res Int. 2024 Feb 6;32(48):27572–89. doi: 10.1007/s11356-024-32241-0 (PMC12695975; doi:10.1007/s11356-024-32241-0)
Supplement: Supplementary file 1 — Supplementary file1 (DOCX 114 KB) [file 11356_2024_32241_MOESM1_ESM.docx]

# **Supplementary material**

# **Assessment and comparison of thermochemical pathways for the rice residues valorization: pyrolysis and gasification**

Myriam Quintero-Naucil^1^, Jairo Salcedo-Mendoza^1^, Juan Camilo Solarte-Toro^2^, Valentina Aristizábal-Marulanda^1,3*^

^1^ Facultad de Ingeniería, Grupo Procesos Agroindustriales y Desarrollo Sostenible (PADES), Universidad de Sucre, Sincelejo - Colombia

^2^ Grupo de investigación en Procesos Químicos, Catalíticos y Biotecnológicos, Instituto de Biotecnología y Agroindustria, Universidad Nacional de Colombia – Sede Manizales, Manizales - Colombia

^3^ Facultad de Tecnologías, Escuela de Tecnología Química, Grupo de Investigación en Desarrollo de Procesos Químicos, Universidad Tecnológica de Pereira, Pereira – Colombia

^*^Corresponding author: [valentina.aristizabal2@utp.edu.co](mailto:valentina.aristizabal2@utp.edu.co), Carrera 27 #10-02 Álamos – Block 6, Zip code: 660003

**Table 1S.** Equation used to calculate the kinetic parameters of the FWOD, OFW and KAS models.

| **Model** | **Equation** | **Axis X** | **Axis y** | **Slope** | **Reference** |
| --- | --- | --- | --- | --- | --- |
| OFW | $Log\left( \beta\right)=Log\left( \frac{A*Ea}{R*f\left( \alpha\right)} \right)-2.315-\frac{0.467*Ea}{RT}$ | 1/T | $Log \beta$ | $\frac{0.467*Ea}{R}$ | (Jai Bhagwan Dahiya, Krishan Kumar 2006) |
| FWOD | $Ln\left( \beta\right)=Const-1.052\left( \frac{Ea}{RT} \right)$ | 1/T | $Ln\left( \beta\right)$ | $1.052\left( \frac{Ea}{R} \right)$ | (Joseph H. Flynn 1966), (Ozawa 1965) |
| KAS | $Ln\left( \frac{\beta}{T^{2}} \right)=Ln\left[ \frac{A*Ea}{Ea*f(\alpha)} \right]-\left( \frac{Ea}{RT} \right)$ | 1/T | $Ln\left( \frac{\beta}{T^{2}} \right)$ | $\frac{Ea}{R}$ | (Kissinger 1957) |
| **Preexponential factor** | | | | | |
| Vyazovki method | $A=B_{i}*E_{a}\frac{e^{(\frac{E_{a}}{R*Tm})}}{R*T^{2}}$ | | | | (Koga et al. 2023) |

**Section 1: Calculation of adiabatic flame temperature**

Equation 1 shows the stoichiometric balance for complete biomass combustion.

$CH_{x}O_{y}N_{z}+ m\left( O_{2}+3.76N_{2} \right)\to{CO}_{2}+ H_{2}O+ N_{2}$ **Eq. 1**

Where x, y, z come from the empirical formula based one carbon atom of the biomass and m is the amount of stoichiometric air necessary for complete combustion of the biomass.

Assuming thar the combustion gases are a mixture of ideal gas, it is solved as shown in **Equatio 2** as follows.

$\int_{298}^{T} \bar{C}_{p}dt=aT+ \frac{bT^{2}}{2}+ \frac{cT^{3}}{3}+\frac{dT^{4}}{4}$**Eq. 2**

Where a, b, c, d are the species-specific coefficients, shown in the **Table A-2**.

**Table A-2** Cengel 7^th^ edition (Yunes A Cengel 2005)

| **Gas species** | **a** | **b** | **c** | **d** | **Temperature range (K)** |
| --- | --- | --- | --- | --- | --- |
| CO_2_ | 22,26 | 5,98X^-02^ | -3,50X^-05^ | -7,47X^-09^ | 273-1800 |
| H_2_ | 29,11 | -1,92X^-03^ | 4,00X^-06^ | -8,70X^-10^ | 273-1800 |
| CO | 28,16 | 1,68X^-03^ | 5,37X^-06^ | -2,22X^-09^ | 273-1800 |
| CH_4_ | 19,89 | 5,20X^-02^ | 1,27X^-05^ | -1,10X^-08^ | 273-1800 |
| H_2_O vapor | 32,24 | 1,92X^-03^ | 1,06X^-05^ | -3,60X^-09^ | 273-1800 |
| N_2_ | 28,9 | -1,57X^-03^ | 8,08X^-06^ | -2,87X^-09^ | 273-1800 |

The adiabatic flame temperature is approximately 580°C for rice husk and rice straw.

**Table 2S.** Mass and energy indicators, price of raw materials, utilities and products.

| **Mass indicators** | **Equation** | | **Reference** |
| --- | --- | --- | --- |
| Product Yield ($Y_{p}$) | $\text{Y}_{\text{P}}\text{ = }\frac{{\dot{\text{m}}}_{\text{Produc, i}}}{{\dot{\text{m}}}_{\text{OPW}}}$ | | (Solarte-Toro 2022) |
| Annual production ($P_{p}$) | $\text{P}_{\text{P}}\text{ =} M_{p}* W_{p}$ | | (Piedrahita-Rodríguez et al. 2022) |
| Carbon conversion efficiency (CCE) | $\text{CCE = }\frac{C in product (mol)}{C in biomass (mol)}$ | | (Aristizábal-Marulanda et al. 2021) |
| Lower heating value of syngas (LHV) | $LHV=12.74*Y_{H_{2}}+39.82*Y_{{CH}_{4}}+12.63*Y_{{CH}_{4}}$ | | (Basu 2013) |
| Cold gas efficiency (CGE) | $CGE=\frac{{\dot{m}_{syngas}*LHV}_{syngas}}{\dot{m}_{biomass}*{LHV}_{biomass}}$ | | (Dhanavath et al. 2018) |
| **Energy indicator** | **Equation** | | Reference |
| Specific energy consumption ($S_{EC}$) | $\text{S}_{\text{EC}}\text{ = }\frac{\dot{Q}\text{ + }\dot{W}}{\dot{m}_{OPW}}$ | | (Aristizábal-Marulanda et al. 2021) |
| Overall energy efficiency ($\text{η}$) | $\text{η =}\frac{\dot{m}_{product}\text{* }\text{LHV}_{product}}{\left( \dot{m}_{OPW}\text{* }{LHV}_{OPW} \right)\text{ + }\dot{Q}\text{ + }\dot{W}}$ | | (Aristizábal-Marulanda et al. 2021) |
| Resource energy efficiency $(\eta_{E})$ | $\eta_{E}\text{ }\text{=}\frac{\left( \dot{m}_{energy vector}\text{* }{LHV}_{energy vector} \right)}{\dot{m}_{biomass}*{LHV}_{biomass}}$ | | (Solarte-Toro 2022) |
| **Component** | **Value** | **Unit** | **Reference** |
| Rice husk or rice straw | 46.85 ^a^ | USD/ton | This work |
| Cooling water | 1.78 ^a^ | USD/cum | This work |
| Electricity | 0.15 ^a^ | USD/KWh |  |
| Bio-char | 50 | USD/ton | (Beston Group 2023) |
| Bio-oil | 130 | USD/ton | (Tewfik et al. 2009) |
| Electricity | 0.15^a^ | USD/KWh | This work |

$\dot{m}_{product}:$is the mass flow of the product, [kg/h]. ${\dot{\text{m}}}_{\text{OPW}}$ is the mass flow of the raw material, [kg/h]. $M_{p}$ is the mass of product in a year, [ton/year]. $Wp$: specific period of the process. $\sum\dot{m}_{inputs}$: mass flows of process input streams, [kg/h]. $\sum\dot{m}_{output}:$ mass flows of process output stream, [kg/h]. $\dot{\text{Q}}$ are the energy (heat) requirements of the process, [MJ/h]. $\dot{\text{W}}$ are the energy (electricity) requirements of the process, [MJ/h]. $LHV$:Lower heating value, [MJ/Kg] or [MJ/Nm^3^]. $\dot{m}_{energy vector}:$is the mass flow of the energy vector, [kg/h]

^a^ Price for the Department of Sucre

**Table 3S.** GWP values for different time horizons (IPCC 2007)

| **Gas** | **20 years** | **100 years** | **500 years** |
| --- | --- | --- | --- |
| **Carbon Dioxide** | 1 | 1 | 1 |
| **Methane** | 72 | 25 | 7.6 |
| $\mathbf{Climate change=}\sum{GWP}_{a,i}*m_{i}$ | | | |

${GWP}_{a,i}$: is the Global Warming Potential for substance *i* integrated over years. $m_{i}$: [Kg] is the quantity of substance *i* emitted.

**Table 4S.** FTIR spectrum band assignments.

| **Wavenumber (cm^-1^)** | **Funtional groups** | **Compounds** | **Reference** |
| --- | --- | --- | --- |
| 3600-3000 | O-H stretching | Carbohydrates (hemicellulose, cellulose)  Phenols (lignin)  Alcohols  Carboxylic acid | (Kumar et al. 2020), (Le Troedec et al. 2008), (Thakur et al. 2018) |
| 3000-2800 | C-H*_n_* stretching of aliphatic groups | Polysaccharides  Aromatic  Aliphatic | (Yang et al. 2007), (Le Troedec et al. 2008) |
| 2929.34 | Asymmetric C-H stretching present in alkyl groups such as methyl or methylene group | Hemicellulose  Cellulose | (El-Hendawy 2006) |
| 2856.06 | Symmetric C-H stretching present in alkyl groups such as methyl or methylene group | Hemicellulose  Cellulose | (El-Hendawy 2006) |
| 1716.32 | C=O stretching of acetyl group | Hemicellulose | (Choudhary et al. 2022), (Le Troedec et al. 2008) |
| 1639.2 | C-C stretching | Hemicellulose  Lignin | (Kumar et al. 2020) |
| 1513.85 | C=C stretching of the aromatic ring skeleton of phenylpropane | Lignin | (Alriols et al. 2009) |
| 1344.14 | C-H stretching of phenolics groups | Lignin | (Choudhary et al. 2022) |
| 1228.43 | Si-O-Si functional group | Silice | (Choudhary et al. 2022), (Kumar et al. 2020) |
| 1200 – 1000 | C-O stretching in lignin and xylan.  C-H stretching of cellulose and hemicellulose | Hemicellulose  Cellulose  Lignin | (Choudhary et al. 2022) |
| 867.81 | β-glycosidic | Amorphous cellulose | (Choudhary et al. 2022) |

# **References**

Alriols MG, Tejado A, Blanco M, et al (2009) Agricultural palm oil tree residues as raw material for cellulose, lignin and hemicelluloses production by ethylene glycol pulping process. Chemical Engineering Journal 148:106–114. https://doi.org/10.1016/j.cej.2008.08.008

Aristizábal-Marulanda V, Solarte-Toro JC, Cardona Alzate CA (2021) Study of biorefineries based on experimental data: production of bioethanol, biogas, syngas, and electricity using coffee-cut stems as raw material. Environmental Science and Pollution Research 28:24590–24604. https://doi.org/10.1007/s11356-020-09804-y

Basu P (2013) Biomass Gasification, Pyrolysis and Torrefaction: Practical Design and Theory

Beston Group (2023) Pyrolysis plant. https://www.bestongrupo.es/planta-de-pirolisis/. Accessed 4 Jun 2023

Choudhary M, Jain SK, Devnani GL, et al (2022) Thermal kinetics and morphological investigation of alkaline treated rice husk biomass. Journal of the Indian Chemical Society 99:100444. https://doi.org/10.1016/j.jics.2022.100444

Dhanavath KN, Shah K, Bhargava SK, et al (2018) Oxygen-steam gasification of karanja press seed cake: Fixed bed experiments, ASPEN Plus process model development and benchmarking with saw dust, rice husk and sunflower husk. Journal of Environmental Chemical Engineering 6:3061–3069. https://doi.org/10.1016/j.jece.2018.04.046

El-Hendawy ANA (2006) Variation in the FTIR spectra of a biomass under impregnation, carbonization and oxidation conditions. J Anal Appl Pyrolysis 75:159–166. https://doi.org/10.1016/j.jaap.2005.05.004

IPCC (2007) IPCC Fourth Assessment Report: Climate Change 2007. https://archive.ipcc.ch/publications_and_data/ar4/wg1/en/ch2s2-10-2.html. Accessed 8 Oct 2023

Jai Bhagwan Dahiya, Krishan Kumar MM-H and BH (2006) Kinetics of isothermal and non-isothermal degradation of cellulose: model-based and model-free methods. Polym Int 55:961–969. https://doi.org/10.1002/pi.2398

Joseph H. Flynn LAW (1966) A quick, direct method for the determination of activation energy from thermogravimetric data. Journal of Polymer Science Part C: Polymer Letters. https://doi.org/10.1002/pol.1966.110040504

Kissinger HE (1957) Reaction Kinetics in Differential Thermal Analysis. Guang Pu Xue Yu Guang Pu Fen Xi/Spectroscopy and Spectral Analysis 31:1042–1046. https://doi.org/10.1021/ac60131a045

Koga N, Vyazovkin S, Burnham AK, et al (2023) ICTAC Kinetics Committee recommendations for analysis of thermal decomposition kinetics. Thermochim Acta 719:. https://doi.org/10.1016/j.tca.2022.179384

Kumar M, Mishra PK, Upadhyay SN (2020) Thermal degradation of rice husk: Effect of pre-treatment on kinetic and thermodynamic parameters. Fuel 268:. https://doi.org/10.1016/j.fuel.2020.117164

Le Troedec M, Sedan D, Peyratout C, et al (2008) Influence of various chemical treatments on the composition and structure of hemp fibres. Compos Part A Appl Sci Manuf 39:514–522. https://doi.org/10.1016/j.compositesa.2007.12.001

Ozawa T (1965) A New Method of Analyzing Thermogravimetric Data. Bull Chem Soc Jpn 38:1881–1886. https://doi.org/10.1246/bcsj.38.1881

Piedrahita-Rodríguez S, Solarte-Toro JC, Piñeres PP, et al (2022) Analysis of a biorefinery with multiple raw materials in the context of post-conflict zones in Colombia: plantain and avocado integration in the Montes de María region. Biomass Conversion and Biorefinery 12:4531–4548. https://doi.org/10.1007/s13399-022-02560-8

Solarte-Toro JC (2022) Sustainability assessment of different biorefinery schemes to enhance the development of post-conflict areas in the Colombian context: The Montes de Maria case

Tewfik SR, Sorour MH, Abulnour AMG, et al (2009) Bio-oil from rice straw by pyrolysis: Experimental and techno-economic investigations. 8th World Congress of Chemical Engineering: Incorporating the 59th Canadian Chemical Engineering Conference and the 24th Interamerican Congress of Chemical Engineering 7:59–67

Thakur LS, Varma AK, Mondal P (2018) Analysis of thermal behavior and pyrolytic characteristics of vetiver grass after phytoremediation through thermogravimetric analysis. J Therm Anal Calorim 131:3053–3064. https://doi.org/10.1007/s10973-017-6788-0

Yang H, Yan R, Chen H, et al (2007) Characteristics of hemicellulose, cellulose and lignin pyrolysis. Fuel 86:1781–1788. https://doi.org/10.1016/j.fuel.2006.12.013

Yunes A Cengel MAB (2005) Termodinámica
